# Splanchnic vein thrombosis in myeloproliferative neoplasms: treatment algorithm 2018

**DOI:** 10.1038/s41408-018-0100-9

**Published:** 2018-06-26

**Authors:** Guido Finazzi, Valerio De Stefano, Tiziano Barbui

**Affiliations:** 1 USC Hematology, ASST Papa Giovanni XXIII, Bergamo, Italy; 2 Institute of Hematology, Catholic University, Roma, Italy; 3 IRCCS Policlinico Gemelli Foundation, Roma, Italy; 4 FROM Research Foundation, ASST Papa Giovanni XXIII, Bergamo, Italy

## Abstract

Myeloproliferative neoplasms (MPNs) are a leading cause of splanchnic vein thrombosis (SVT). SVT is observed in all MPNs and frequently affects young patients. Therapy should be addressed to three main goals: preventing thrombosis recurrence, managing the underlying MPN, and supporting liver dysfunction. Life-long oral anticoagulation with vitamin K antagonists is the cornerstone of the antithrombotic treatment. However, recurrences of SVT or other thrombosis may occur in 15–20% of patients. Direct oral anticoagulants can represent an alternative and preliminary data encourage comparative studies. Survival of patients with SVT in MPN is primarily influenced by the natural history of the underlying neoplasms, rather than the SVT event. An aggressive management is recommended and a treatment algorithm based on the different MPN subtypes is proposed. Hydroxyurea is the cytoreductive drug of choice in polycythemia vera and essential thrombocythemia, whereas ruxolitinib is indicated in intermediate and high-risk patients with myelofibrosis and in PV patients resistant or intolerant to hydroxyurea. The management of SVT in MPNs requires a multidisciplinary approach that may include a hematologist, a gastroenterologist, an interventional radiologist, and a surgeon. In the case of clinical deterioration despite pharmacological therapy, patients with SVT should be considered for invasive procedures or liver transplantation.

## Introduction

Splanchnic vein thrombosis (SVT) includes portal vein thrombosis (PVT), mesenteric (MVT) and splenic vein thrombosis, and the Budd–Chiari syndrome (BCS)^1^. BCS is the least frequent manifestation of the SVT spectrum, with an estimated incidence of about 0.5–1 case per million people per year^1^. The incidence of PVT and MVT is reported to range between 0.7/100,000 and 2.7/100,000 person-years^1^. SVT can be defined as “primary” or “secondary”, depending on the presence or absence of associated abdominal or systemic risk factors. Hematologic disorders, autoimmune diseases, and the use of hormonal therapy are the most common risk factors in BCS, whereas liver cirrhosis, abdominal cancer, intraabdominal inflammatory conditions, and surgery are the most common risk factors in PVT/MVT^2–4^.

During the last several years, myeloproliferative neoplasms (MPNs) have emerged as a leading systemic cause of SVT^5^. Results from the largest cohort studies report a prevalence of MPNs of about 10% when all SVT patients are included, and up to 50% in cohorts including patients with BCS only^2–4^. SVT is observed in all MPNs and affects mainly young patients. In one of the largest studies of MPN-associated SVT, 181 cases were retrospectively recruited from 23 European centers: polycythemia vera (PV), essential thrombocythemia (ET), and primary myelofibrosis (PMF) accounted for 37, 37, and 26% of cases, respectively; 65% of the patients were women and median age at diagnosis was 48 years^6^. A similar distribution of SVT in MPNs subtypes was recently reported in a single center study of 84 consecutive cases: 35% were PV, 30% ET, and 35% PMF, respectively;^7^ 67% of the patients were women and median age at diagnosis was 54 years. In a study of 538 MPN patients younger than 40 years, SVT occurred in 26 cases (4.8%) during a median follow-up of 7 years, representing the most frequent incident thrombotic event^8^.

SVT may represents the first clinical manifestation of MPN, particularly when the JAK2V617F mutation is present. The prevalence of JAK2V617F mutation in patients with SVT ranges between 28% in non-malignant, non-cirrhotic patients with PVT to 41% in patients with BCS. JAK2V617F screening in patients with SVT without overt MPN features identified MPN in about 15% of cases^9,10^. These patients are included in the MPN, unclassifiable (MPN-U) category of the current WHO classification, among those MPN-like neoplasms that cannot be clearly classified as one of the other subcategories of MPNs^11,12^.

**Fig. 1:**
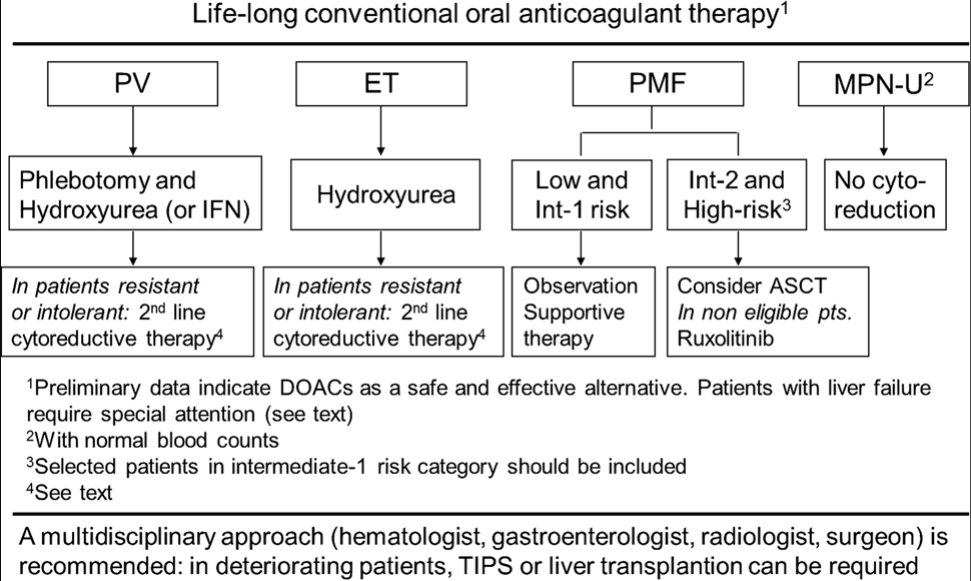
Current treatment algorithm in splanchnic vein thrombosis in myeloproliferative neoplasms. PV polycythemia vera, ET essential thrombocythemia, PMF primary myelofibrosis, MPNu myeloproliferative neoplasms, unclassifiable, IFN interferon, ASCT allogeneic stem cell transplantation, DOACs direct oral anticoagulants, TIPS transjugular intrahepatic portosystemic shunt

Management of SVT in MPN is a clinical challenge because of the frequent young age of the patients and the severity of short-term and long-term outcomes if inadequately treated. Therapy should be addressed to three main goals: (a) preventing recurrences of thrombosis; (b) managing the underlying MPN; and (c) supporting organ dysfunction, particularly of the liver (Fig. 1).

## Antithrombotic treatment

### Conventional anticoagulant therapy

In the acute phase, the antithrombotic treatment of patients with SVT and MPNs does not differ from that of patients without MPNs^5^. They should be treated promptly with full-dose low molecular or unfractionated heparin followed by vitamin K antagonists (VKA), maintaining PT INR in the therapeutic range of 2.0–3.0. Notably, warfarin monitoring can be difficult in patients with BCS and liver failure, due to the reduced production of coagulation factors, altering the baseline INR. The optimal duration of VKA is unknown, but in general a life-long treatment is suggested, considering the presence of a permanent risk factors for thrombosis, such as the underlying MPN^13,14^. In a multicentre prospective registry of SVT patients, anticoagulant drugs are employed in almost the totality of MPN patients^3^, as recommended by the guidelines from the American College of Chest Physicians^15^.

Safety and efficacy of long-term VKA therapy for MPN patients with SVT have been evaluated in some studies. In a retrospective cohort of 44 patients with PVT and MPN (median follow-up 5.8 years), 21 (48%) received long-term therapy: 9 with VKA, 6 with VKA plus aspirin, and 6 with aspirin. Recurrent thrombotic events occurred in 12 patients (27%), 9 in the absence of antithrombotic prophylaxis and 3 during VKA treatment. During follow-up, 17 patients (39%) experienced at least one episode of gastrointestinal bleeding^16^. A weak efficacy of VKA has been reported in a series of 36 BCS patients who had recurrent thrombosis after orthotropic liver transplantation (OLT) in 42% of cases, with no difference in the mean INR between patients who developed thrombosis and patients who did not (2.73 vs. 2.70, *p* = 0.47)^17^. In the aforementioned series of 181 patients with MPNs and SVT, recurrent thrombotic events were recorded in 31 patients (17%), including SVT in 45% of the recurrent events, during a median follow-up of 3.2 years. Reported risk factors for SVT recurrence included history of thrombosis, BCS, splenomegaly, and marked leukocytosis. The rate of recurrences per 100 pt-years with or without VKA was 3.7 (95% CI: 2.3–5.5) and 7.2 (95% CI: 3.1–14.3), respectively, with only a trend in favor of anticoagulation (*p* = 0.09)^6^. More recently, the Mayo clinic experience with 84 consecutive cases of SVT in MPN patients was reported: 14 patients (16%) had recurrent vascular events, including 8 (10%) with recurrent SVT. Post-SVT thrombosis-free survival and recurrence rate were not affected by type of SVT or initial treatment strategy^7^. Thus, several studies failed to convincingly demonstrate the therapeutic value of conventional systemic anticoagulation, showing a non-trivial rate of event recurrence. Accordingly, it is reasonable to consider alternative forms of anticoagulation, such as the use of low molecular weight heparin or direct-acting oral anticoagulants.

### Low molecular weight heparin

Current guidelines recommend LMWH for the treatment of cancer-associated venous thromboembolism (VTE)^15,18^, on the basis of their superior safety and efficacy compared to VKA, as demonstrated by the CLOT and CATCH trials^19,20^. Given the neoplastic nature of MPN, such an approach could be attractive also in these patients. However, published evidence about efficacy and safety of long-term administration of LMWH in MPN is lacking, since, while the CLOT and CATCH trials did include cases with hematologic malignancies, this comprised only ~10% of the population of patients in either trial. In addition, at variance with most cancers, the MPN disease activity persists for decades, so that continued life-long treatment with daily s.c. heparin can be troublesome. LMWH is the drug of choice for MPN patients with SVT during pregnancy and puerperium.

### Direct oral anticoagulants (DOACs)

Direct inhibitors of thrombin (dabigatran) or factor Xa (rivaroxaban, apixaban) present practical advantages over VKA, such as the administration in fixed doses without need of systematic laboratory control and a minor interaction with food and drugs. Most importantly, clinical trials and meta-analyses showed that DOACs are as effective as VKA in preventing recurrent VTE and might be associated with a lower incidence of major, particularly cerebral, hemorrhages^21^. In clinical practice, the use of DOACs is expanding also for the treatment of VTE in cancer^22,23^ and in in atypical locations, such as SVT, although the reported data are still limited.

The largest series hitherto published include 70 patients with SVT from 17 centers. Thirty-six patients (38%) had cirrhosis. DOACs used were rivaroxaban (83%), dabigatran (11%), and apixaban (6%). Patients were followed-up for a median duration of 15 months (cirrhotic) and 26.5 months (non-cirrhotic). Adverse events occurred in 17% of patients and included one case of recurrent PVT and five cases of bleeding. Treatment with DOACs was stopped in three cases^24^. Another study compared three groups of patients: (a) 63 patients with VTE in atypical locations (AL, 26 with SVT) treated with DOACs (rivaroxaban and apixaban), (b) 23 patients with VTE in AL (22 with SVT) treated with enoxaparin, and (c) 352 patients with VTE in typical locations treated with DOACs. Recurrence rate for the VTE-AL group receiving DOAC was 7.3 per 100 person-years, which was not different when compared with those of the other groups. Major bleeding rate in the VTE-AL group receiving DOACs was 7.2 per 100 person-years, not different compared with those of the other groups^25^.

Patients with BCS and hepatic failure pose a number of specific challenges regarding the choice of the optimal DOAC, since dabigatran generally does not require dose modification, but also has increased risk of gastro-intestinal bleeding (therefore to be avoided in patients with varices related to liver disease), apixaban is held only for Child-Pugh class C, and rivaroxaban/edoxaban are held for Child B and C.

These data suggest that DOACs are effective and safe in patients with DVT in atypical locations, including SVT, and provide a basis for performing randomized clinical trials of DOACs vs. low molecular weight heparin or VKA.

## MPN subtype-adapted treatment algorithm

Various studies in MPN-associated SVT reported that survival was primarily influenced by the expected natural history of the underlying MPN, rather than the SVT event per se. Hoekstra et al. reported that 12 of 44 patients (27%) had progression of the underlying MPN after a median follow-up of 5.8 years. Seventeen patients (39%) died at a median age of 64 years. Death was directly related to end-stage MPN in eight patients (47%) and to a new thrombotic event in three case (18%)^16^. In the aforementioned study from Mayo Clinic, 22 deaths and 3 leukemic transformations were recorded at a median follow-up of 2.7 years^7^. The cause of death was liver failure in three individuals (all PMF), upper GI bleed in one (PMF), leukemic transformation in one (PMF), and thrombosis in one (ET), while it was felt to be unrelated to SVT in 12 instances and unknown in 4. As expected, post-SVT survival was significantly shorter in patients with PMF, compared to those with PV or ET (HR 4.5, 95% CI: 1.9–10.9). Other significant risk factors for overall survival, in multivariable analysis, included older age (HR 5.0, 95% CI: 1.6–14.5) and abnormal cytogenetics (HR 3.9, 95% CI: 1.4–11.3). These observations emphasize the need of an aggressive management of the underlying MPN in patients with SVT.

### Polycythemia vera

PV patients with SVT are classified at high risk of thrombosis recurrence, also if they young of present with only a moderate increase of hemoglobin or hematocrit (“masked” PV)^8^. Thus, cytoreductive drug therapy is indicated, in addition to phlebotomy, with the goal to maintain hematocrit below 45%^26^ and, possibly, platelet count ≤400 × 10^9^/L and WBC count <10 × 10^9^/L^27^. According to current recommendations^28^, either hydroxyurea or rIFNα is the first-line cytoreductive therapy at any age. Among the two, our preferred choice is hydroxyurea. Notably, HU may recognize additional antithrombotic mechanisms of action besides pan myelosuppression, including qualitative changes in leukocytes, decreased expression of endothelial adhesion molecules, and enhanced nitric oxide generation^29^. In one of the few studies that addressed the issue of recurrent thrombosis in PV and ET, cytoreduction with hydroxyurea in 77% of cases halved the risk of recurrence^30^. However, in the specific setting of MPN-related SVT cytoreductive treatment has not been proven to be effective in reducing the rate of recurrence. In the European series of 181 SVT patients, in those receiving cytoreduction 23 recurrent events were recorded over 537 pt-years (IR 4.2 per 100 pt-years) and in patients without cytoreductive treatment 8 recurrent events were recorded over 198 pt-years (IR 4.0 per 100 pt-years)^6^. Similarly, no benefit from cytoreduction in preventing recurrences was observed in the Mayo cohort^7^. In another recent study of 597 MPN patients with transient ischemic attack or ischemic stroke (IS), cytoreductive therapy with hydroxyurea in about 80% of cases was a strong protective factor for recurrent IS (HR: 0.24), but not for venous thrombosis^31^.

In PV patients resistant or intolerant to HU^32^, both rINFα and ruxolitinib are appropriate second-line drug therapies^28,33–36^.

### Essential thrombocythemia

Current guidelines favor hydroxyurea as first-line therapy of ET patients in need of cytoreduction, including those with SVT^28^. Anagrelide or rINFα are the recommended second-line therapy in patients resistant or intolerant to hydroxyurea^28,34,37–39^. In a randomized phase II trial of ruxolitinib vs. best available therapy (BAT) in ET patients resistant or intolerant to HU, no evidence of improvement in complete response and in the rates of thrombosis, hemorrhage, and transformation were observed^40^. However, some disease-related symptoms improved in patients receiving ruxolitinib relative to BAT, as reported also in another long-term, uncontrolled phase II study^41^. Thus, at variance of PV, ruxolitinib is not indicated in ET patients resistant or intolerant to HU, with the possible exception of those severely symptomatic, particularly for pruritus^42^.

### Primary myelofibrosis

Current prognostication systems in myelofibrosis are based on the risk of mortality rather than of thrombosis^43–45^. Thus, the presence of SVT does not change the risk classification of PMF patients and SVT can be observed in all risk categories.

In asymptomatic patients with low or intermediate-1 risk disease according to IPSS/DIPPS/DIPSS plus classifications, there is no evidence to support the value of disease-modifying therapy and observation alone is recommended^28^. Some of these patients might require palliative therapy for anemia, splenomegaly, or constitutional symptoms^28^. If cytoreductive treatment for the reduction of leukocytosis or thrombocytosis is indicated, the first-line drug of choice is hydroxyurea^28^. Ruxolitinib is recommended as first-line approach in PMF patients with intermediate-2 or high-risk disease, not candidates to allogeneic stem cell transplantation (ASCT)^28,46–49^. This drug is also indicate in PMF patients with intermediate-1 disease when splenomegaly or systemic symptoms in need of treatment are present^28,49,50^.

Ruxolitinib has been specifically evaluated in 21 patients with MPN-associated SVT in a phase 2 clinical trial^51^. The majority of case were diagnosed with PMF (12), and the others with PV (5 cases) and ET (4 cases). Eighteen patients had spleno-portal-mesenteric thrombosis, two had BCS, and one had both sites involved. Ruxolitinib was well tolerated with hematological toxicities consistent with those of patients without SVT. After 24 weeks of treatment, spleen volume reduction >35% by MRI was achieved by 29% of patients, MPN-related symptoms, evaluated by dedicated questionnaires, improved significantly during treatment. Treatment with ruxolitinib did not produce meaningful improvements in the status of esophageal varices, but was associated with substantial stabilization of varices grade at week 72, as observed in 85% of the patients; furthermore there was only one bleeding episode. This compares favorably with a prospective study in non-cirrhotic, non-tumoral PVT, where probability of worsening of existing esophageal varices at 1 year was 13%^52^. Overall, the trial showed that ruxolitinib is safe in patients with MPN-associated SVT, and effective in reducing spleen size and disease-related symptoms.

ASCT is recommended for all eligible PMF patients with IPSS/DIPSS/DIPSS-plus high or intermediate-2 risk and in selected cases with intermediate-1 risk^28,53^. A careful evaluation of patient performance status and additional comorbidities is critical in the assessment of transplant candidacy. The impact of SVT has not been specifically studied, but the presence of portal hypertension and severe splenomegaly may increase the risk of graft failure and early post-transplant hepatotoxicity^54^. However, despite these caveats, SVT does not represent an absolute contraindication to ASCT in patients with MPNs.

### MPN, unclassifiable

The need for cytoreduction in patients with SVT carrying the JAK2V617F mutation who present with normal blood counts and do not meet the diagnostic criteria for MPN is uncertain^5^. Approximately half of patients will not develop an overt MPN during the follow-up^9^ and the clinical course of most of these patients is indolent;^55^ accordingly, there is no evidence to prescribe cytoreductive regimens^5^. An expert panel identified the appropriateness of myeloproliferation-directed therapy in patients with SVT-associated MPN as an unmet clinical need and provided methodological suggestions to tackle this issue^56^.

## Liver function support

### Invasive procedures

The management of SVT in MPNs requires a multidisciplinary approach that may include a hematologist and/or a thrombosis expert, a gastroenterologist/hepatologist, and an interventional radiologist and/or a surgeon. In the case of clinical deterioration despite anticoagulation, cytoreductive therapy and supportive care, including sodium restriction, diuretic therapy and paracentesis, patients with BCS should be considered for invasive procedures, such as angioplasty with or without stenting, transjugular intrahepatic portosystemic shunt (TIPS), or surgical portosystemic shunt^13^.

In patients with short segment stenosis or occlusion of the hepatic veins with significant patent segments, it is desirable to overcome the obstruction between hepatic vein remnants and the inferior vena cava by means of balloon angioplasty with or without stenting. This approach will re-establish hepatic venous outflow via the physiological route. Use of thrombolysis may enhance the success rate of these procedures. After failure of angioplasty or stenting, a surgical portosystemic shunt or TIPS should be considered. The rationale for surgical portosystemic shunting is to convert the portal vein into an outflow tract of the liver, so that patients with severe forms of BCS might benefit from decompression of the liver^13^.

### Liver transplantation

OLT should be considered as effective treatment for rapidly progressive BCS after failure of conventional treatment or portosystemic shunting, occurring in 10–20% of patients^3^. In a retrospective cohort of 78 BCS patients, there was no significant difference between the MPN (*n* = 41) and the non-MPN (*n* = 37) groups with regard to long-term survival. Twelve of the 41 MPN patients (29%) died within the first 3 years after OLT, but death was related to the hematologic disease only in one case with recurrent BCS. Among survivors, no progression to myelofibrosis or acute leukemia was observed during a mean follow-up time of 12.4 years (range 3–28.4 years)^57^, confirming the absence of MPN evolution reported in a series of 17 BCS patients even at long-term follow-up of up to 20 years after OLT^58^.

## Conclusions

Similarly to the non-MPN patients with SVT, long-term anticoagulation with VKA is recommended in patients with MPN-related SVT. However, in this setting the efficacy of treatment in preventing recurrences is not clearly demonstrated and there is room to plan clinical trials aimed to investigate alternative antithrombotic strategies, such as DOACs.

Indication to cytoreductive treatment is adapted from the guidelines concerning the different MPN subtypes, but there is no strong evidence that is of benefit in preventing recurrent thrombosis after MPN-related SVT. Preliminary results in preventing worsening of esophageal varices have been reported in patients treated with ruxolitinib, but further investigations are needed in this field.

In conclusion, long-term treatment of MPN-related SVT remains an unmet clinical need; to date, the indication to long-term anticoagulation and cytoreduction is not specifically evidence-based, but derives from the benefits observed either in the non-MPN patients with SVT and in the MPN patients with thrombosis of other sites.
